# Tolerance of chronic HDACi treatment for neurological, visceral and lung Niemann-Pick Type C disease in mice

**DOI:** 10.1038/s41598-018-22162-7

**Published:** 2018-03-01

**Authors:** Md. Suhail Alam, Bruce Cooper, Joseph D. Farris, Kasturi Haldar

**Affiliations:** 1 Boler-Parseghian Center for Rare and Neglected Diseases, University of Notre Dame, Notre Dame, IN 46556 USA; 2 Department of Biological Sciences, University of Notre Dame, 103 Galvin Life Sciences, Notre Dame, Notre Dame, IN 46556 USA; 3 Bindley Bioscience Center, Discovery Park, Purdue University, West Lafayette, IN 47907 USA

## Abstract

Histone deacetylase (HDAC) inhibitors are of significant interest as drugs. However, their use to treat neurological disorders has raised concern because HDACs are required for brain function. We have previously shown that a triple combination formulation (TCF) of the pan HDACi vorinostat (Vo), 2-hydroxypropyl-beta-cyclodextrin (HPBCD) and polyethylene glycol (PEG) 400 improves pharmacokinetic exposure and entry of Vo into the brain. TCF treatment significantly delayed both neurodegeneration and death in the *Npc1*^*nmf164*^ murine model of Niemann-Pick Type C (NPC) disease. The TCF induces no metabolic toxicity, but its risk to normal brain functions and potential utility in treating lung disease, a major NPC clinical complication, remain unknown. Here we report that TCF administered in healthy mice for 8–10 months was not detrimental to the brain or neuromuscular functions based on quantitative analyses of Purkinje neurons, neuroinflammation, neurocognitive/muscular disease symptom progression, cerebellar/hippocampal nerve fiber-staining, and *Hdac* gene-expression. The TCF also improved delivery of Vo to lungs and reduced accumulation of foamy macrophages in *Npc1*^*nmf164*^ mice, with no injury. Together, these data support feasibility of tolerable, chronic administration of an HDACi formulation that treats murine NPC neurological disease and lung pathology, a frequent cause of death in this and possibly additional disorders.

## Introduction

Histone deacetylase inhibitors (HDACi) are emerging therapeutics for a broad range of diseases including cancer and neurodegeneration^1–4^. They block HDAC enzymes, to promote acetylation of both histones and non-histone proteins to elicit complex cellular changes^5,6^. HDACi-induced histone modifications have been shown to directly increase or decrease transcriptional expression of the mutated target gene(s) in many genetic diseases as well as transduce indirect beneficial effects through modulation of chaperone and proteostatic networks^7–9^. Due to their broad effects on transcription, it is particularly important to maximize HDACi efficacy while limiting dose. We previously reported on development and validation of a therapeutic strategy of a triple combination formulation (TCF) of the HDACi vorinostat (Vo) that enabled us lowering Vo dose to treat cerebral disease, as well as inflammation in liver and spleen, in a mouse model of a fatal cerebellar disorder Niemann-Pick Type C (NPC)^10^.

NPC is caused by a defect in either the *Npc1* or *Npc2* genes^11^. It is a rare autosomal recessive neurodegenerative disease. Approximatley, 95% of cases are due to a defect in *Npc1*. Cells with defects in either *Npc* genes accumulate cholesterol in late endosomes/lysosomes^12,13^. A point mutation in the *Npc1* gene that blocks cholesterol transport in cells is causative for neurodegeneration in a mouse model^14^. At the organismal level, in the central nervous system (CNS), *Npc1* is essential for neuronal function^15,16^. Neurodegeneration is a hallmark of clinical NPC disease. Disease progression can be heterogeneous and slow, but once initiated, it is invariably fatal^11^. Splenomegaly and hepatomegaly are common presenting symptoms in pediatric cases followed by neurocognitive and neuromuscular degeneration^17^. Lung disease is also prominent and can even be the cause of death^18,19^.

Presently the only available treatment for NPC is miglustat (Zavesca™), an iminosugar that decreases glycosphingolipid accumulation in type1 Gaucher’s disease^20,21^. It was approved for NPC treatment in Europe, Canada and Japan but denied FDA approval (although it is prescribed off label in the US). Miglustat may confer a mild improvement in specific clinical symptoms, but fails to prevent disease progression^22,23^. 2-Hydroxy propyl beta cyclodextrin (HPBCD) is being investigated as an emerging therapy^24,25^. It chelates cholesterol but does not cross the blood brain barrier (BBB)^26^. Therefore, to treat neurological disease HPBCD must be directly delivered to the CNS^27,28^ which carries procedural risk for life-long therapy. Systemic delivery is needed to improve liver and other visceral organs but inexplicably, HPBCD is excluded from lung^29,30^ and therefore it is of little benefit to end-stage, advanced and frequently fatal bronchial disease. Arimoclomol is another emergent therapy for NPC^31^, but its benefit for systemic disease especially in the treatment of lung inflammatory disease remains unknown.

The TCF combined HPBCD, PEG and Vo in a defined formulation^10^. Upon systemic injection, it increased the plasma exposure of Vo and boosted its delivery across the BBB to stimulate histone acetylation in the brain. Importantly, mice chronically treated for close to a year showed no metabolic toxicity^10^. However, the effects of long-term TCF exposure on key neurons, brain areas and overall progression of symptoms of neurodegeneration, as well as inhibition of *Hdac* genes known to be important for brain function, remain unknown. Further, while HPBCD reduces systemic inflammation^10,24,29^, it is excluded from lungs^29,30^ and therefore whether the TCF promotes Vo delivery and therapeutic action in lungs remains unidentified. Our findings on these points advance the pre-clinical development of a new HDACi therapeutic strategy to treat NPC and other difficult-to-treat disorders that may benefit from epigenetic therapy.

## Methods

### Materials

All fine chemicals including 2-hydroxypropyl-*β*-cyclodextrin (HPBCD) and polyethylene glycol 400 (PEG) were procured from Sigma (St Louis, MO, USA) unless otherwise indicated. Vorinostat (Vo) was from Selleck Chemicals (Houston, TX, USA).

### Animals

*Npc1*^*nmf164*^ is a Balb*/c* strain that carries a D1005G (A to G at cDNA bp 3163) mutation in the *Npc1* gene^32^. A breeding pair of mutant mice were obtained from Robert P. Erickson, University of Arizona Health Sciences Center, Tucson, AZ, USA and is available at ‘The Jackson Laboratories.’ Homozygous mutants (*Npc1*^*nmf164*^) along with wild type (wt) littermates (*Npc1*^+/+^), were generated in house by crossing heterozygous mutant (*Npc1*^+/*nmf164*^) males and females and genotyped as previously described^10^. Wt Balb/*c* mice were procured from Envigo (Indianapolis, IN, USA). All animal experimental protocols were approved by the Institutional Animal Care and Use Committee of the University of Notre Dame and were carried out in accordance with guidelines and regulations provided.

### Drug injection and organ harvest

The triple combination formulation (TCF) is a mixture of Vo (50 mg/kg), HPBCD (2000 mg/Kg)), PEG 400 (45%) and DMSO (5%). Vo (50 mg/Kg) was made in 5% DMSO and 45% PEG. HPBCD was a 20% (w/v) solution and given dose of 2000 mg/Kg. Detailed methodology for preparing drug solutions has been described earlier^10^. To enable comparative studies with prior regimens, all mice were given two doses of HPBCD at 7 and 15 days of age (also referred to as P7 and P15). From P21 onwards, mice received either HPBCD alone or TCF, as indicated. Vo was also initiated at P21. For lung histopathology, *Npc1*^*nmf164*^ mice were analyzed at 100–109 days of age. Long-term safety was assessed for 8–10 months in either healthy *Npc1*^+/*nmf164*^ or commercially purchased wt Balb/*c* mice. Healthy *Npc1*^+/*nmf164*^ mice received two doses of HPBCD (2000 mg/Kg) at 7 and 15 days of age. Starting from 21 days, mice were given weekly TCF injections. Wt mice received weekly injections of TCF starting at 30 days of age without prior HPBCD administration. Injections were administered weekly through the intraperitoneal (i.p) route, and the injection volume used was 10 ml/Kg body weight across all treatment groups. The animals were sacrificed by asphyxiation using CO_2_ and their organs were harvested. A part of the harvested organ was cut and flash frozen in liquid nitrogen and stored at −80 °C to enable additional analyses. The remainder was immersed fixed in 10% neutral buffered formalin (~4% formaldehyde) for 24 hours at RT and subsequently stored in 70% alcohol until transfer to paraffin.

### Histological assessments of brain and lung

Paraffin-embedded sections (4–5 µm) were dewaxed in xylene and alcohol. For Nissl, brain sections were stained with acidified 0.1% cresyl violet for 7 min followed by two incubations in 95% ethanol for 5 min each. The sections were cleared in xylene and mounted in cytoseal XYL (Thermo Scientific, Kalamazoo, USA). H&E staining of brain and lung tissues was carried out according to standard methods^33^. Brain sections were additionally stained with silver ions by a modified Bielschowsky method^34^. Lung sections were also stained with May-Grunwald followed by Giemsa stain^35^. Except for Nissl, all chemical staining was performed by AML laboratories (Saint Augustine, FL, USA). Images were visualized with A10 PL 10×/0.25 or DPIan Apo 40×/1.00 oil immersion objective lens (Nikon) and captured on a Nikon Olympus microscope using a Nikon digital DS-Fi1-U2 camera controlled by NIS-Elements F3.0 Nikon software (all from Nikon Instruments INC, Tokyo, Japan).

### Iba1 immunostaining of brain sections

Paraffin-embedded brain sections (4–5 µm) were dewaxed in xylene and alcohol. Iba1 antigen was retrieved by boiling the sections in acidic condition for 30 min. Blocking was done with 2% goat serum for 30 min at RT. Sections were incubated with anti-Iba1 (1:500, 019–19741, Wako Chemicals) overnight at 4 °C. FITC-conjugated secondary IgG antibodies (MP Biomedicals, Solon, OH, USA) were used at a dilution of 1:200. Nuclei were stained with DAPI (0.5 µg/ml) and mounting was done using Vectashield (Vector laboratories). Sections were visualized with 40× oil-immersion objective lens (NA 1.35) and image collection was performed using an Olympus IX inverted fluorescence microscope and a Photometrix cooled CCD camera (CH350/LCCD) driven by DeltaVision software from Applied Precision (Seattle, WA, USA). DeltaVision software (softWoRx) was used to deconvolve these images. Images are single optical sections. Images were analyzed using ‘softWoRx’ or ‘ImageJ’ software (NIH, MD, USA).

### RNA extraction and quantitative PCR

Total RNA from the brain of 6 untreated and 6 TCF-treated, 8-month old wt mice was extracted using RNeasy Plus Universal kit from Qiagen. The RNA was quantified by Nanodrop 2000 (Thermo Fisher Scientific, Waltham, MA, USA). Quantitative PCR (qPCR) was performed using Power SYBR Green RNA-to-CT 1-Step Kit and an ABI Prism 7500 Fast real-time PCR system (Applied Biosystems, Grand Island, USA). The reaction was set in 20 μl using 100 nM primers and 200 ng total RNA as a template, in triplicate wells. The thermal cycling parameters were as follows: step 1, 48 °C for 30 min; step 2, 95 °C for 10 min; step 3, 95 °C for 15 sec; step 4, 60 °C for 15 sec. Step 3–4 was repeated for 40 cycles followed by melt curve analysis. The nucleotide sequences of the primers were, for *Hdac3*, forward, 5′-CCCGAGGAGAACTACAGCAG-3′; reverse, 5′-ACTCTTGGGGACACAGCATC-3′, for *Hdac4*, forward, 5′-TCTGCCAAATGTTTTGGGTA-3′; reverse, 5′-TCACAGATGGCTGTCAGGTC-3′, for *Hdac*7, forward, 5′-TGTCCAGACTCCTGGCTACC-3′; reverse, 5′-CATGGGTTCTTCCTCTTCCA-3′, for *Gapdh* (*Glyceraldehyde 3-phosphate dehydrogenase*), forward, 5′-TCCATGACAACTTTGGCATTG-3′; reverse, 5′-CAGTCTTCTGGGTGGCAGTGA-3′. Specific amplification was validated by melt curves and agarose gel electrophoresis. For each gene, reactions were set in triplicate wells. One *Hdac* gene along with *Gapdh* was tested per plate. The relative quantification of gene expression was done by the *Δ*CT method. Briefly, the fold change in the transcript level for a particular gene in an individual well was calculated by dividing the CT (Cycle Threshold) value by the mean CT of 6 untreated mice.

### Quantification of Vo in lungs

*Npc1*^+/*nmf164*^ mice (age 6–7 weeks) were given intraperitoneal injections of either Vo (50 mg/Kg in 45% PEG and 5% DMSO) or TCF (Vo, 50 mg/Kg + HPBCD, 2000 mg/Kg + PEG, 45% + DMSO, 5%). At 30 min and 1 h post injection, mice were asphyxiated with CO_2_, blood was drawn by cardiac puncture and organs were perfused with 20 ml ice-cold PBS through the ventricle. Harvested lungs were cut into small pieces (4–6 mm^2^) and flash frozen in liquid nitrogen. The quantification of Vo was done by Metabolite Profiling Facility, Bindley Bioscience Center, Purdue University, IN, USA: detailed methods are as described earlier^10^. Briefly, the tissue was homogenized using a Precelly bead homogenizer system utilizing ceramic CK 14 beads. 2 ng of deuterated internal standard (d_5_-Vo, Toronto Research Chemicals, Ontario, Canada) was added to lung homogenate prior to liquid extraction with acetonitrile. Prior to analysis, samples were reconstituted in 100 µL of 50% water/50% acetonitrile. An Agilent 1200 Rapid Resolution liquid chromatography (HPLC) system coupled to an Agilent 6460 series triple quadrupole mass spectrometer (MS/MS) was used to analyze Vo. The data were obtained in positive electrospray ionization (ESI) mode and quantitated by monitoring the following transitions: for Vo, 265→232 with a collision energy of 5 V and for d_5_- Vo, 270→237 with a collision energy of 5 V.

### Statistical Test

Two tail Student’s *t* test or Mann-Whitney U test were carried out to determine the statistical significance of the data. p < 0.05 was considered significant.

## Results

### Assessment of chronic TCF-treatment in cerebellar and hippocampal regions as well as neurobehavioral/cognitive disease in mice

HDACs are important enzymes and their functions are required in brain development^36–38^. In particular, HDAC3 knockdown blocks development of Purkinje neurons^39^. It has therefore been hypothesized that long-term HDACi treatment may adversely affect the brain. However, we previously reported that weekly administration of the TCF in *Npc1*^*nmf164*^ mice prevented loss of Purkinje cell neurons^10^. Since Vo from the TCF peaked at 30 minutes post injection and was rapidly cleared from the brain and plasma^10^, we hypothesized that epigenetic modulation associated with a transient block of HDAC3 (as well as other HDACs) may be well tolerated and benefit NPC-diseased animals.

**Figure 1:**
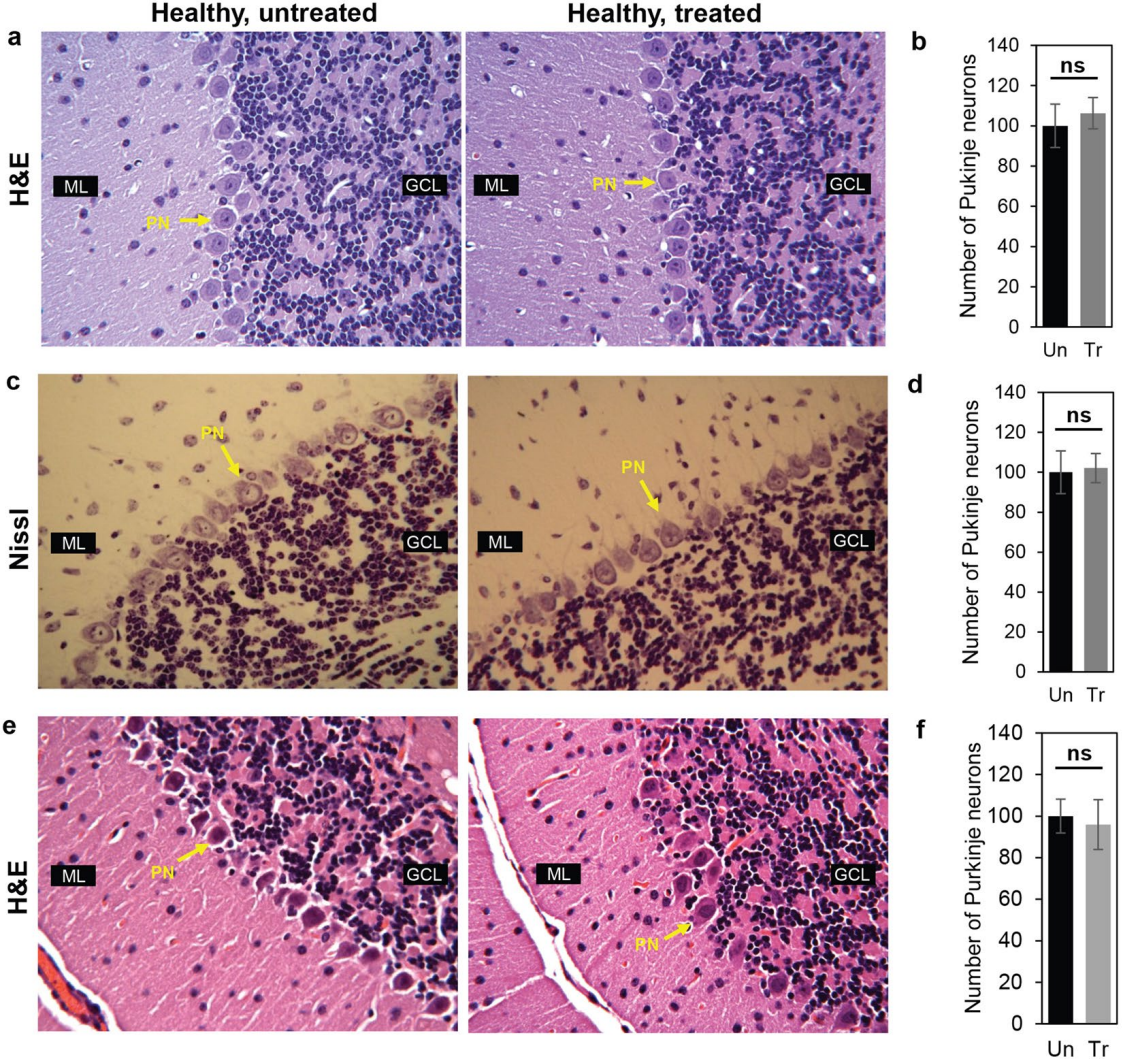
Analysis of Purkinje neurons in healthy mice after long-term TCF-treatment. (**a**–**d**) H&E and Nissl stained micrographs and quantitative analyses of cerebellum from untreated (108 days) and TCF-treated healthy (*Npc1*^+/*nmf164*^, 225–265 days) mice. Treated mice were initially given HPBCD at 7 and 15 days of age. An injection of TCF was administered at day 21 and then weekly up to 8–10 months. Purkinje neurons are indicated by arrows. Representative images are presented; n = 4 were examined for every condition. (**b and d**) Quantification of Purkinje neurons in cerebellum from six mice in treated and untreated groups. Two sections per mouse were analyzed. **(e)** H&E stained micrographs of cerebellum from 8 months old untreated and TCF-treated wt healthy mice, where animals were given a weekly injection of TCF starting from 30 days of age without pretreatment with HPBCD prior to weaning. Representative images from 6 untreated and 8 treated mice are shown. **(f)** Quantification of Purkinje neurons from six mice in untreated and treated groups. Images were taken with a 40x objective. Numbers (mean ± SD) in treated (Tr) are expressed as percent relative to comparator untreated (Un) mice. GCL, Granule Cell Layer; ML, Molecular Layer; PN, Purkinje Neurons; ns, non-significant.

But the effects of long term exposure of TCF on neurons in the normal animal brain, remain unaddressed. Since Purkinje are major neurons requiring HDAC function, we used them as a sentinel neuron for the effects of extended TCF-treatment in healthy animals. We administered weekly TCF to heterozygous, healthy ‘control’ animals for 2–3 fold longer (240–300 days) than the 100 day-efficacy period established in *Npc1*^*nmf164*^ mice. As shown in Fig. 1a,b, H&E staining failed to show any change in histological features or quantitation of Purkinje neurons in the cerebellum upon chronic TCF-treatment relative to untreated animals. Nissl staining, which primarily marks the rough endoplasmic reticulum and is an indicator of active neurons, suggested that Purkinje neurons are intact and present in the same numbers in chronic TCF-treated compared to untreated mice (Fig. 1c,d).

Mice in Fig. 1a–d were pre-treated with HPBCD at days 7 and 15 days followed by TCF treatment initiated post weaning at 21 days. This is because in prior work^10^, we wanted to compare the relative benefit of TCF to the maximal benefit possible by HPBCD alone (for which HPBCD treatment has to be initiated at P7). To rule out the possibility that early administration of HPBCD may confer protection against later toxicity caused by TCF, we initiated administration of TCF in wt Balb/*c* mice starting at 30 days, without prior HPBCD administration. Subsequently, TCF was chronically administered for up to 8 months of age and as shown by H&E staining, treated animals showed no significant difference in the histology or numbers of Purkinje neurons compared to untreated controls (Fig. 1e,f). Together the data in Fig. 1 indicate that even though recurrent exposure to low concentration of Vo via the TCF is sufficient to trigger sustained epigenetic effects^10^ it does not cause neuronal loss despite prolonged times of treatment (up to 8 months).

**Figure 2:**
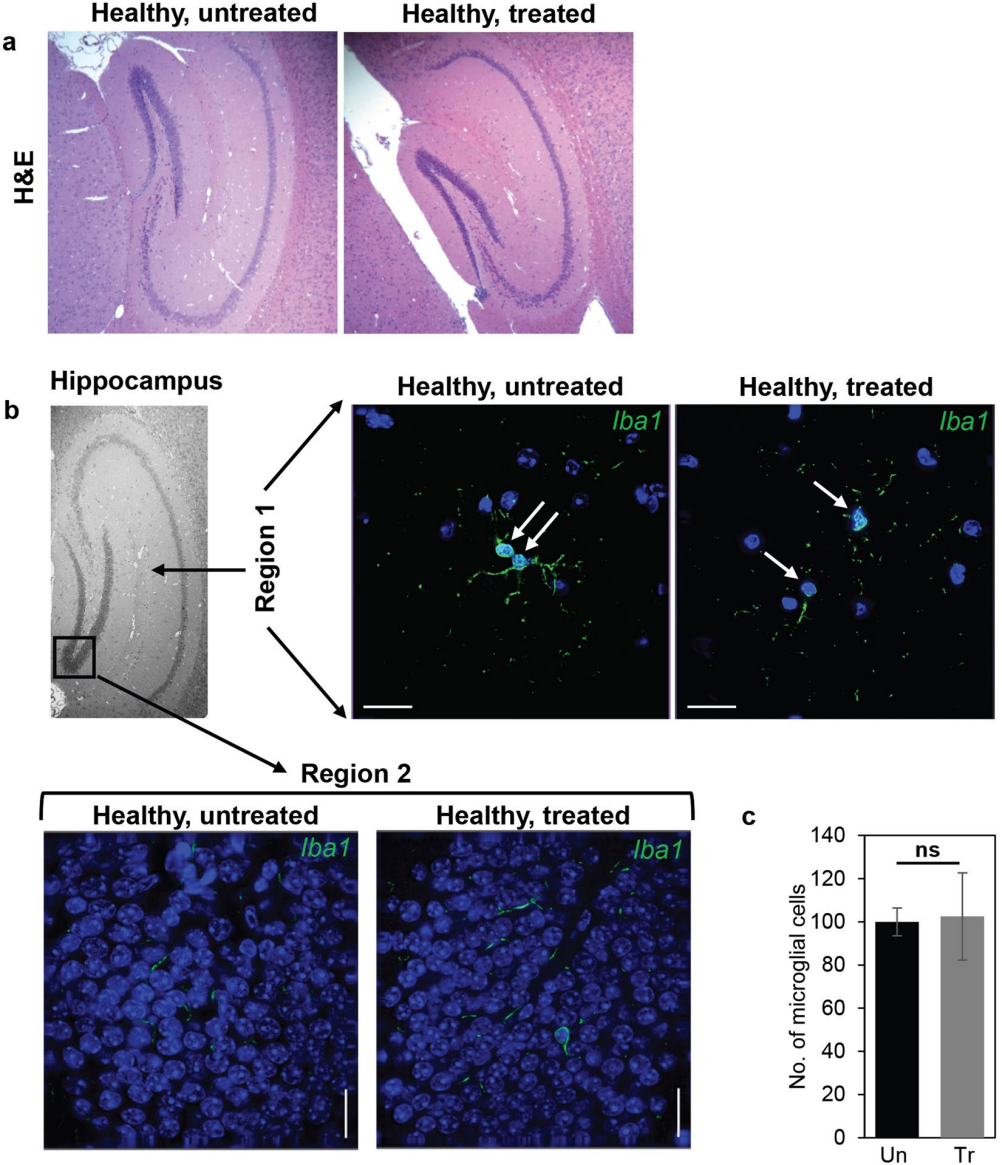
Assessment of the hippocampus of healthy mice after chronic, long-term TCF treatment. (**a**) Histological analysis using H&E staining and **(b)** indirect immunofluorescence analyses of activated microglia in the hippocampus of untreated and TCF-treated healthy (*Npc1*^+/*nmf164*^) mice. In panel b, anti Iba1 antibodies were used to detect activated microglial cells (green signal, white arrows, from the regions indicated). Treated mice were given two doses of HPBCD at 7 and 15 days of age followed by weekly injection of TCF from 21 days of age. The images shown are representative from two untreated (age 108 and 109 days) and four TCF-treated (age 225–265 days) healthy mice, **(c)** Quantification of Iba1+ microglial cells from six mice per group (2 sections per mouse). Numbers (mean ± SD) are presented at percent change compared to untreated healthy mice. Scale bar = 25 µm. ns, non-significant.

We additionally examined the effects of chronic TCF treatment on the hippocampus. As shown in Fig. 2a, normal mice exposed to weekly TCF treatment for 225–265 days showed comparable hippocampal morphology detected by H&E staining relative to untreated animals at 100–110 days. When we tracked activation of microglial cells by staining for Iba1 (which marks inflammation), only a few resident microglia were seen in the hippocampus of both TCF-treated and untreated animals (Fig. 2b). This is in contrast to our finding of Iba1 stained microglial accumulation in the hippocampus of *Npc1*^*nmf164*^ mice which was alleviated by weekly TCF treatment^10^. Quantitative analysis of the data in Fig. 2b, showed no significant difference in the number of microglial cells in untreated and chronically TCF-treated mice (Fig. 2c). This suggested that even though Vo is predicted to transcriptionally activate numerous target genes, the TCF does not induce an inflammatory response damaging to neurons.

Clinically, NPC disease is defined by major and minor symptomatic domains whose severity has been quantified to monitor the natural history of the disease^40^. While awaiting plasma biomarkers^41–44^, symptom scoring continues as an important index of progressive disease. We previously created a disease severity scale for murine NPC that captures major patient disease domains scored in a defined range and whose sum provides a cumulative disease score, (with a maximal score of 13)^10^. Because older healthy animals, particularly males, often displayed poor grooming and slight impairment in limb tone onwards of 100 days, a cumulative score of 3 or higher reliably flags the onset of symptomatic disease. We have previously shown that while untreated *Npc1*^*nmf164*^ mice progress to a score of 10–13 by 100 days, the TCF affords significant reduction to a score of 4–5 when administered over the same period^10^, rendering functional benefit to major symptomatic domains of neurological disease (that include ambulation, cognition, motor control and dysphagia). When this score was applied to healthy animals treated for 100 days, the cumulative score remained below the baseline.

**Figure 3:**
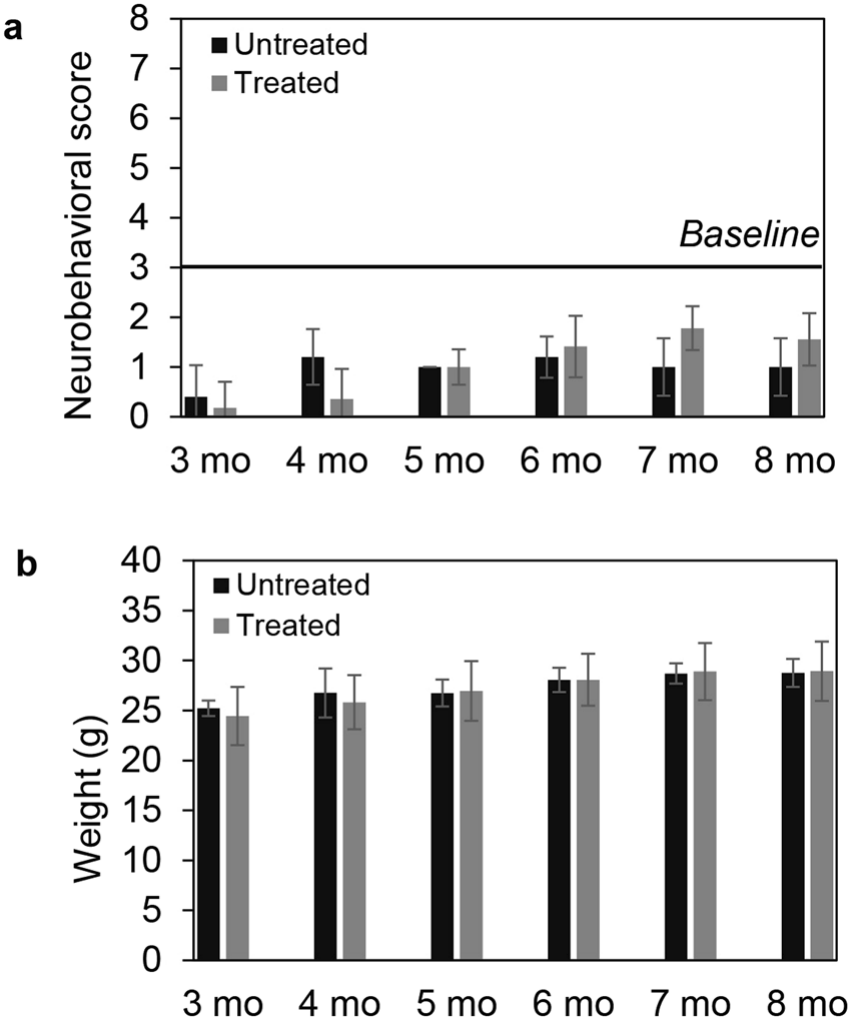
Assessment of chronic TCF treatment on neurobehavioral/cognitive disease scores and body weight in healthy mice. Mice (wt) were given a weekly injection of TCF starting at 30 days of age (without prior administration of HPBCD in the pre-weaning period) upto a maximum of 8 months of age. 15 untreated males and 17 treated males and females (7 males and 10 females) were monitored at 3 to 6 months of age. 7 untreated males and 9 treated (4 male and 5 female) mice, were assessed till 8 months **(a)** The following major neurobehavioral symptoms (and corresponding human disease domain) tremor (motor), gait (ambulation), grooming (cognition), body position (cognition and motor), limb tone (motor) were assessed on a scale of 0–2. Weight loss (dysphagia) was assessed on a scale of 0–3. A score of 0 indicates an asymptomatic state whereas a score of 2 (or 3 for weight loss) indicates a severely symptomatic condition. The cumulative score is shown at the indicated time points. A cumulative score of 3 or below is considered baseline. (**b)** Weight of mice in (a). Numbers are mean ± SD. See also Supplementary Table S1.

Here, we show that when healthy animals were chronically treated with once-a-week TCF for 8 months, the cumulative scores continued to remain below 3 (Fig. 3a, Supplementary Table S1). Notably these animals were treated with TCF starting at 30 days of age without pre-weaning treatment with HPBCD (Fig. 3a, Supplementary Table S1). Scores of 1–2 were observed in animals chronically treated with TCF, but this was also seen in untreated animals and associated with poor grooming in older mice. TCF-treatment induced no change in animal weight (Fig. 3b) suggesting it did not impair overall nutrient consumption and utilization (which marks mid- and end-stage neurological disease)^10^.

### Chronic TCF administration does not reduce quantitative staining of nerve fibers or transcript levels of *Hdac* genes

**Figure 4:**
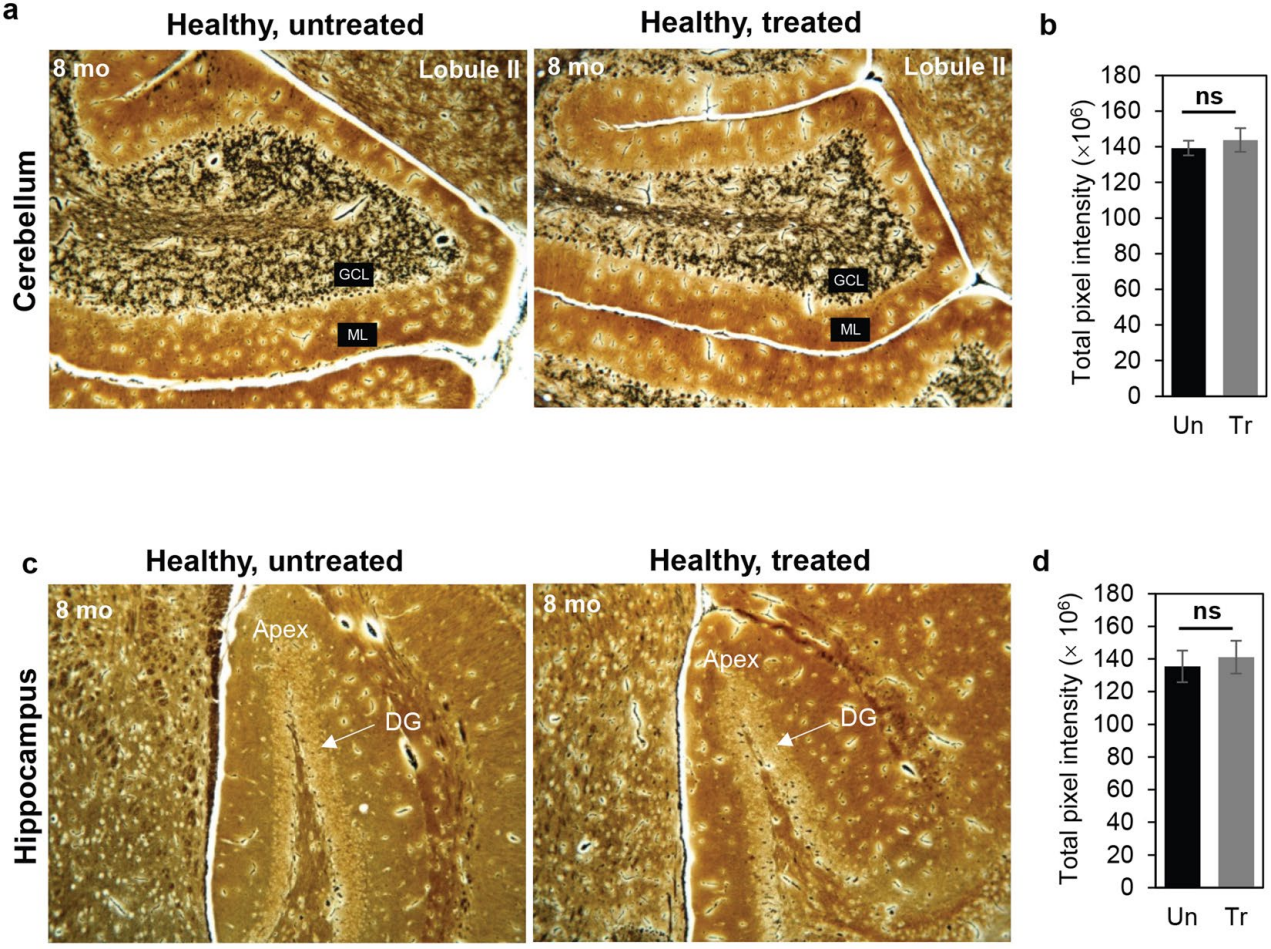
Histological analysis of cerebellum and hippocampus by Bielschowsky stain in 8 month old TCF treated healthy mice. Mice (wt) were given a weekly injection of TCF starting at 30 days of age (without prior administration of HPBCD in the pre-weaning period) and sacrificed at 8 months. **(a**,**b)** Cerebellar micrographs corresponding to the second lobule and their quantitative analyses. **(c**,**d)** Hippocampal images show the dentate gyrus (DG) and their quantitative analyses. In **a**,**c**. representative images (taken with the 10 × objective) from 6 untreated and 8 treated mice are shown. In **b**,**d** for six mice in each group, total pixel intensity was quantified using ImageJ. Numbers are mean ± SD. GCL, Granule Cell Layer; ML, Molecular Layer; Un, untreated; Tr, treated. ns, non-significant.

Our analyses in Figs 1–2 suggested that neuronal cell bodies were not diminished by TCF-mediated chronic HDACi over 8 months. To examine the effects on nerve fibers, we stained sections with silver. As shown in Fig. 4 in both the cerebellum and hippocampus, the intensity of nerve fiber staining was the same in mice chronically treated with TCF compared to untreated animals, providing additional evidence to support that long-term administration of TCF did not adversely affect the neurons.

**Figure 5:**
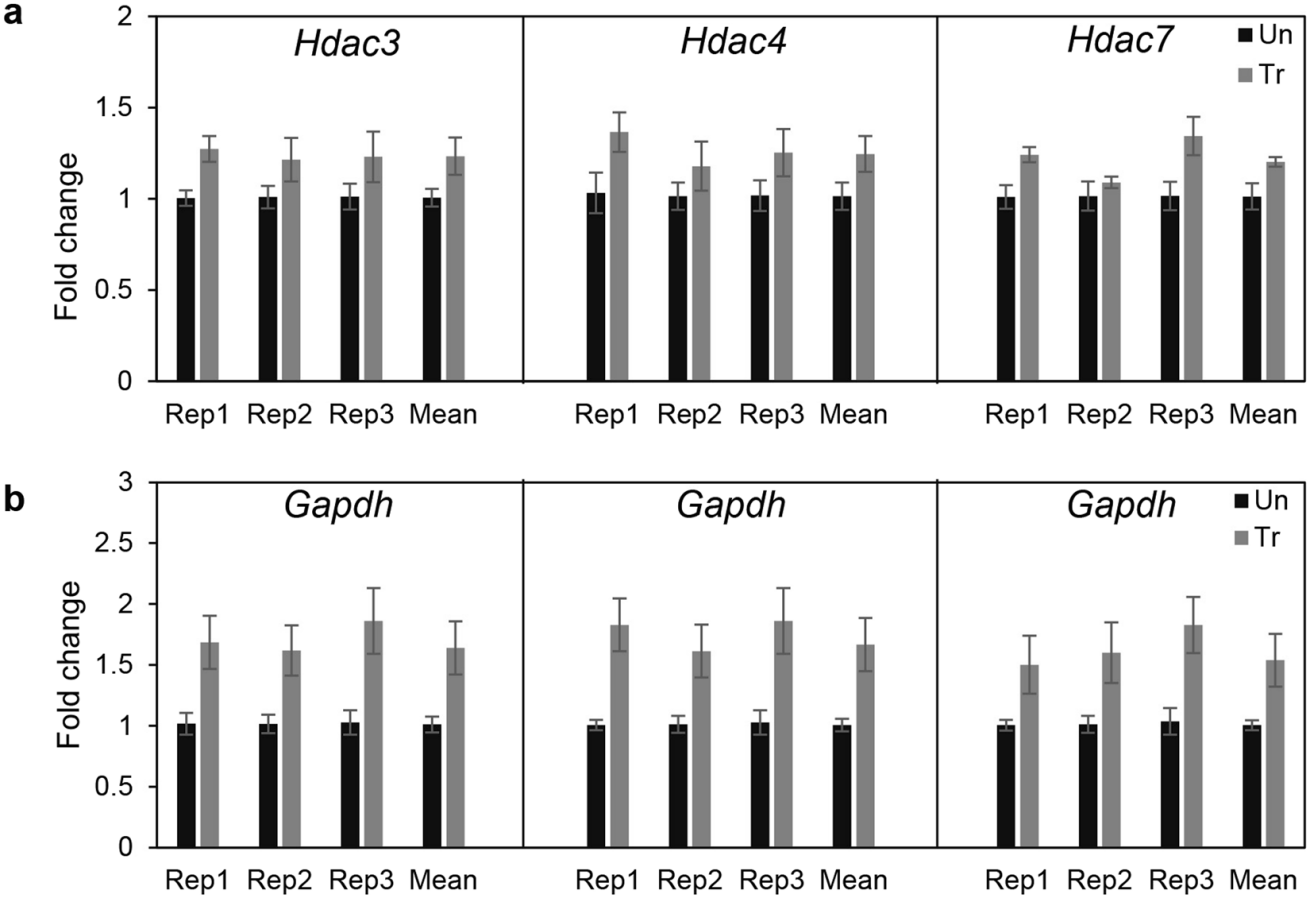
Transcriptional analysis of HDAC genes by qPCR in the brain of healthy mice treated up to 8 months of age. Mice (wt) were given a weekly injection of TCF starting at 30 days of age (without prior administration of HPBCD in the pre-weaning period), sacrificed at 8 months of age and RNA was analyzed from the brain of six untreated (un) and six treated (Tr) mice. Three replicates (Rep1, Rep2 and Rep3) of fold change seen in (**a)** *Hdac3*, *Hdac4* and *Hdac7* and **(b)** *Gapdh*. The fold change in transcript level was calculated by dividing the CT (Cycle Threshold) value by the mean CT of untreated mice. Transcript levels of *Hdac* genes in treated (Tr) mice (grey bars) were comparable to those in untreated (Un, black bars) counterparts. Transcript levels of *Gapdh* appeared to show a modest increase (<2 fold) in treated mice. All values are mean ± SEM.

Finally, we examined the effects of chronic TCF administration on *Hdac* expression, since diminishing *Hdacs* may be detrimental to brain function^38,45^. In particular, *Hdac3* is known to be essential for Purkinje neurons and brain function; indeed its knock down causes severe neurodevelopmental abnormalities and premature death^36,39^. *Hdac4* is essential for synaptic plasticity, spatial learning and memory^46^ whereas *Hdac7* protects neurons from apoptosis^47^. Although most *Hdacs* play a role in the brain^38,45^, functions controlled by *Hdac3, 4* and *7* are crucial. Since Vo is broad acting, enzymatic activities of HDAC3, 4 and 7 are likely to be blocked by the TCF. However, our prior pharmacokinetic studies^10^ showed that >90% of Vo was cleared from the brain 2 hours post injection of TCF. Therefore, subsequent expression of new *Hdac* transcript would likely be sufficient to restore their native function but whether this could be achieved despite chronic weekly administration of the TCF was unknown. We therefore used quantitative PCR to study the expression profile of *Hdac3*, *Hdac4* and *Hdac7* as sentinel markers in the brain of wt mice that received weekly TCF up to 8 months of age. As shown in Fig. 5a, we found no reduction in the transcript level of *Hdac3*, *Hdac4* or *Hdac7* in replicate analyses. There was some increase in the expression of *Gapdh* gene (Fig. 5b). Therefore, rather than normalizing to endogenous *Gapdh* transcript level, we used the *Δ*CT method (see *Methods*) to quantify changes in the transcript levels of these genes from six independent mice. Remarkably, these data revealed that a weekly regimen of chronic administration of TCF does not induce transcriptional repression of key *Hdac* genes.

### TCF increases Vo levels in lungs

 In prior work, we found that 1 h after injection of TCF, Vo concentrations in mouse plasma were 3 fold higher compared to the levels observed when Vo was administered in PEG alone^10^. Vo levels in the brain were also significantly boosted in TCF-injected mice^10^. These data suggested that the HPBCD was a major contributor to the pharmacokinetic (PK) effect in plasma and brain. Further examination of brain, liver and spleen suggested the TCF could treat both neurological as well as systemic NPC disease in mice. However, since HPBCD is known to be excluded from lungs^29,30^, it remained unclear whether the TCF increased exposure of Vo and/or benefit lung disease.

**Figure 6:**
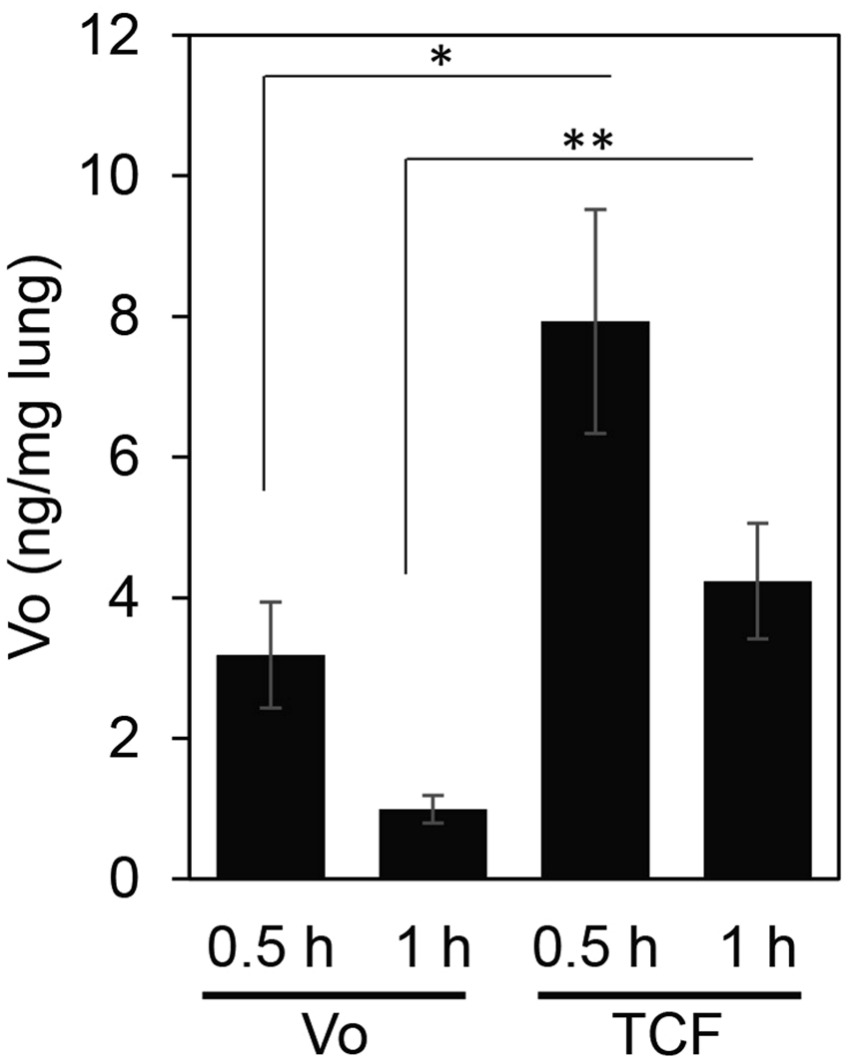
Increased lung concentration of Vo in TCF injected mice. Healthy mice (*Npc1*^+/*nmf164*^) were i.p. injected with Vo alone or TCF. At the indicated times following drug treatment, animals were sacrificed and perfused with PBS. Harvested lungs were homogenized and Vo concentration was determined by mass spectrometry. n = 5. h, hour. *p = 0.02, TCF *vs* Vo 0.5 h, and **p = 0.014 TCF *vs* Vo, 1 h, two-tailed Student’s *t* test.

As shown in Fig. 6, animals injected with Vo in PEG alone showed a mean concentration of 3.2 ng/mg Vo in lungs at 30 minutes, which decreased to 1 ng/mg by 60 min. After TCF injection, Vo concentration reached 7.9 ng/mg at 30 min and then declined to 4.2 ng/mg at 60 min. These data suggested that the TCF boosted Vo entry into lungs, likely due to the (2.5-3 fold) plasma pharmacokinetic effect (previously reported in ref.^10^). Vo concentrations (of 4.2 ng/mg) detected at 60 min in TCF-treated animals were reduced by 45–50% from levels seen at 30 min. Animals injected with Vo in PEG showed a 65–70% reduction over the same period, suggesting that in addition to boosting peak concentrations, the TCF may also slow down Vo clearance from lungs. Both effects may increase levels and exposure of Vo in lungs.

### TCF reduces the accumulation of foamy macrophages in the lungs of *Npc1*^*nmf164*^ mice

Previous studies^24,29^ have shown that the systemic delivery of HPBCD in NPC mice fails to alleviate lung disease (because HPBCD may not be able to reach the tissue). Since HPBCD is a major component of the TCF, this raised a question on whether the formulation could alleviate lung disease even as it boosted Vo delivery to other organs. To test this, we undertook a histochemical analysis of lungs from control and treated mice. Animals were examined at 100 days of age, since in prior work with the *Npc1*^*nmf164*^ mouse model, we established that this is a time of significant neurological disease, responsive to treatment by TCF.

**Figure 7:**
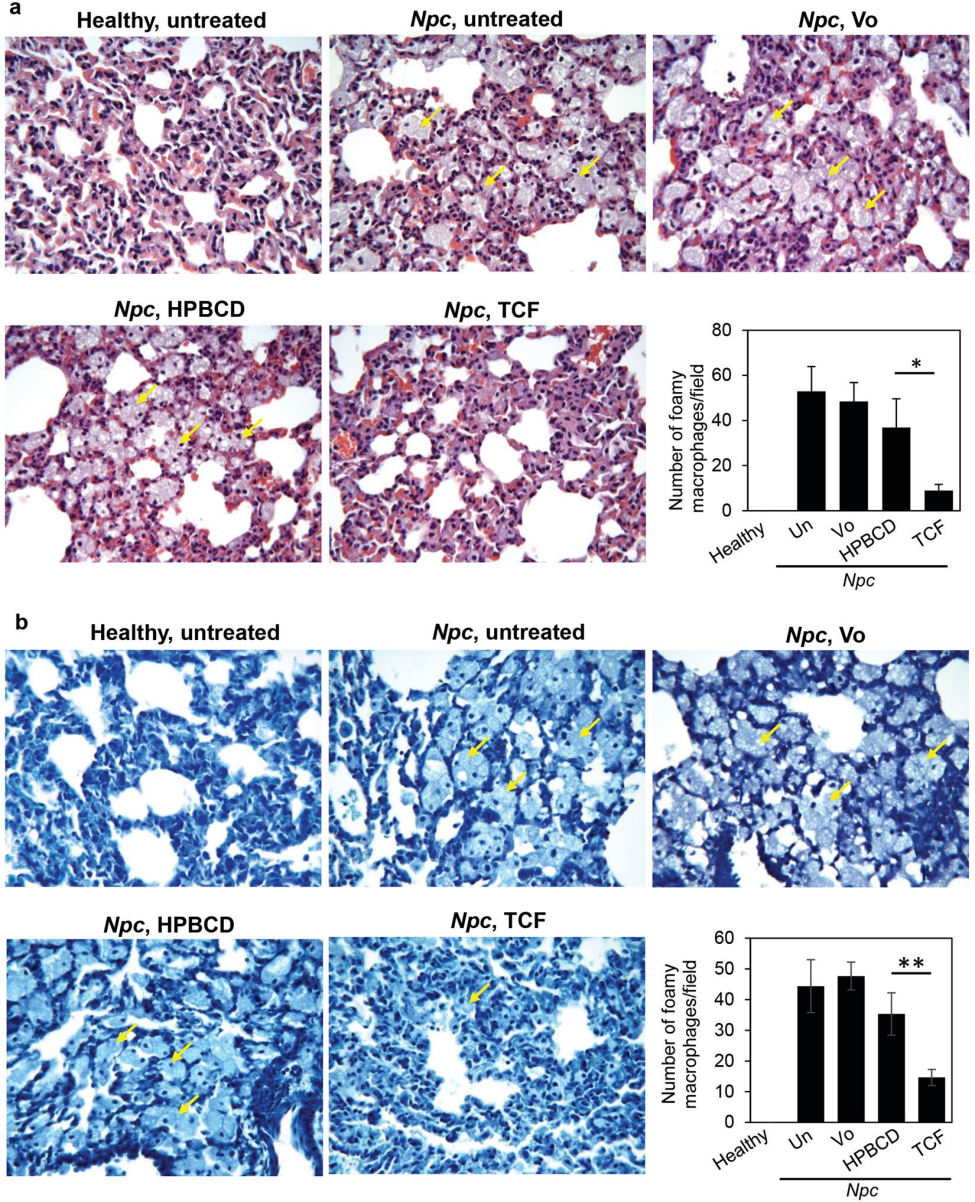
Efficacy of the TCF in reducing accumulation of foamy macrophages in lungs of *Npc* mice. (**a**) H&E and **(b)** May-Grunwald/Giemsa stained micrographs. Foamy macrophages (indicated by arrows) seen at 100 days of age in lungs of *Npc1*^+/*nmf164*^ (healthy control) and *Npc1*^*nmf164*^ (*Npc*) mutant mice with or without chronic TCF-treatment. Foamy macrophages were abundant in untreated *Npc* mice. Treatment with Vo (vorinostat) or HPBCD had no effect whereas TCF treatment significantly reduced the accumulation of foamy macrophages. Images were taken with 40x objective lens and are representative of 4–5 mice in each group. For quantitation, the number of foamy macrophages were counted from randomly selected 10–15 fields per mouse. The data (mean ± SEM) from 4–5 mice in each group are shown in bar diagrams. Un, Untreated.*p = 0.02, **p = 0.03, TCF *vs* HPBCD, two-tailed Mann-Whitney test.

As shown in Fig. 7a, H&E stained micrographs revealed the accumulation of a large number of foamy macrophages in the lungs of untreated *Npc1*^*nmf164*^ mice at 100 days. Semi-quantitative analysis revealed TCF treatment significantly reduced the number of macrophages (Fig. 7a). In contrast, administration of Vo alone or HPBCD continued to be associated with abundant macrophage accumulation (Fig. 7a). Additional analysis of lungs stained with May-Grunwald followed by Giemsa also revealed a reduction in the number of foamy macrophages in TCF treated *Npc1*^*nmf164*^ mice, whereas HPBCD or Vo-alone injected mice showed macrophage accumulation similar to untreated animals (Fig. 7b). Again, semi-quantitative analysis revealed a significant reduction in the accumulation of foamy macrophages in TCF-treated mice (Fig. 7b). Together, these findings suggest that TCF by increasing Vo delivery to lungs, reduced inflammatory pathology there.

### Long-term chronic treatment with TCF shows no deleterious effect on lung histopathology in healthy control animals

**Figure 8:**
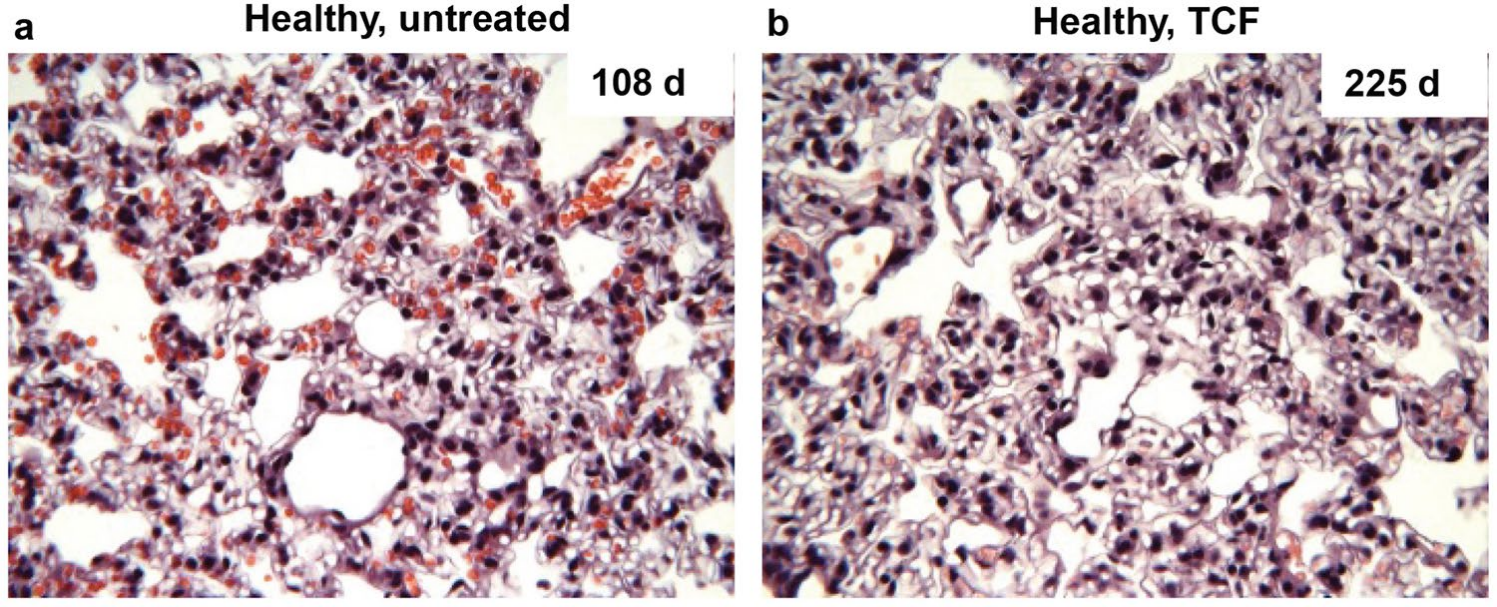
Histological analysis of lungs from long term TCF treated mice. Micrographs show H&E stained sections of lungs from **(a)** untreated and **(b)** weekly TCF-treated healthy (*Npc1*^+/*nmf164*^) mice at 108 and 225 days respectively. No signs of tissue lesions, immune cell invasion or abnormal pathology were seen in long-term TCF-treated mice. The images shown are representative of four untreated mice (harvested at 108–109 days) and four TCF-treated healthy (*Npc1*^+/*nmf164*^) mice (harvested at 225–265 days). Images were taken with the 40x objective lens.

We previously reported that analysis of metabolic markers in the plasma failed to reveal toxicity in liver and kidneys of mice treated once weekly with TCF after 200–300 days^10^. Histological features of liver were also found to be normal^10^. We now report in Fig. 8, that lungs of *Npc1*^+/*nmf164*^ mice at 200–300 days also show the absence of tissue lesions, immune cell invasion or any abnormal pathology (as determined by H&E staining). As a reminder, our prior analyses of treatment efficacy in *Npc1*^*nmf164*^ disease model were undertaken at 100 days. Therefore, the effects of extended TCF administration in healthy *Npc1*^+/*nmf164*^ animals were assessed at 2–3 times longer periods (200–300 days).

## Discussion

Concerns about the intrinsic toxicity of HDACi are pertinent for both pan HDACi and inhibitors designed to target a given HDAC, since even a single HDAC can regulate hundreds of genes (and hence the value of synthesizing selective HDACi has been debated). Since neurological treatments may be long term, it is important to learn the effects of extended treatment periods well beyond when efficacy is detectable, especially in the brain. Our data in Figs 1–4 suggest that the TCF enables chronic administration of a therapeutically viable dose of broad spectrum HDACi with no detectable histological changes in Purkinje neurons and hippocampus as well as neurocognitive/behavioral functions in mice.

Purkinje neurons are major neurons that participate in motor control and learning. They can both emit and receive signals and function to regulate the entire cerebellum. Thus, maintenance of Purkinje cells provides a single readout for not only the complex neuronal process in the cerebellum but also communication from the spinal cord and brain stem. Our data show that the TCF helps preserve of Purkinje cells in the NPC disease model, suggesting these cells are responsive to HDACi (likely due to elevation of NPC1 protein but possibly also by indirect mechanisms). Therefore, our finding that extended exposure to weekly TCF for 8 to 10 months had no effect on Purkinje neuron staining or count, intimates that HDACi administration via the TCF is well-tolerated in Purkinje-associated as well as overall cerebellar functions.

Similarly, the hippocampus, a key region for learning and memory, shows no adverse structural and inflammatory effects despite extended TCF exposure. Although assessment of neurocognitive and behavioral scores do not yield quantitative tissue analyses, they indicate that TCF does not induce symptoms (and therefore processes) of neurodegeneration in wt mice, even though it can delay the appearance of disease processes in the NPC mouse model. Lack of repression of transcription of *Hdac3, Hdac4 and Hdac7* suggests there is no gross inhibition of these key HDAC functions despite chronic TCF treatment, as new HDACs enzymes could potentially be translated from the newly made transcripts. In particular, lack of chronic suppression of *Hdac3* by long-term TCF administration is consistent with the lack of effect of Purkinje neurons. This is likely because the TCF transiently increases low (but transcriptionally active) levels of Vo into the brain. As we have previously shown Vo in the mouse brain is reduced to background levels after 2 h and therefore expected to result in a transient reduction of HDAC3 activity, that may be restored to normal levels in the weekly rest period.

Finally, our findings that the TCF can boost delivery of Vo into lungs and reduce recruitment of macrophages into alveolar spaces, suggests that although HPBCD is excluded, Vo released from the TCF accesses lungs likely due to the plasma exposure. Vo levels delivered to lungs are boosted to sufficiently reduce macrophage levels in *Npc1*^*nmf164*^ mice, which significantly expands the potential of the TCF in treating all organ systems expected to affect the progression of NPC. Extended TCF administration showed no ill-effects on lung pathology of normal mice.

In summary, our current studies suggest that in addition to not stimulating adverse metabolic effects, extended TCF administration failed to induce deleterious responses in brain tissue, overall neurological functions, as well as the lung (although the TCF shows efficacy in all of these domains in the NPC mouse model). This may appear to be counterintuitive, since Vo is a broad acting HDACi at the transcriptional level with potential to target thousands of genes. However, a recent study suggests that proteomic changes may be limited to ~200 targets in NPC diseased cells^48^.

Proteomic analyses of HDACi-induced changes in animal models of disease and health have yet to be undertaken. Moreover, in brain and lung, beneficial effects of Vo are detected in the range of nanogram/mg which are substantially lower than those previously used to study Vo targets in cells and animals. It will be important to establish global transcriptional and proteomic targets of Vo in the 1–10 ng/mg range in both cells and animal organ systems (including the brain). Finally, other than lung, HPBCD and PEG can access all organs in the body cavity and may act to modulate the effects of transcriptional/proteomic changes mediated by Vo primarily by lowering cholesterol and other lipid-related inflammatory pathways, to thereby further enhance long term tolerance of the TCF.

## Electronic supplementary material

Supplementary Table S1
